# A Preclinical Study of Human Embryonic Stem Cell-Derived Mesenchymal Stem Cells for Treating Detrusor Underactivity by Chronic Bladder Ischemia

**DOI:** 10.1007/s12015-021-10204-z

**Published:** 2021-06-29

**Authors:** Hwan Yeul Yu, Jung Hyun Shin, HongDuck Yun, Chae-Min Ryu, Seungun Lee, Jinbeom Heo, Jisun Lim, Juhyun Park, Ki-Sung Hong, Hyung-Min Chung, Dong-Myung Shin, Myung-Soo Choo

**Affiliations:** 1 Department of Urology, Asan Medical Center, University of Ulsan College of Medicine, 88 Olympic-ro 43-gil, Songpa-gu, Seoul, 05505 South Korea; 2 Department of Biomedical Sciences, Asan Medical Center, University of Ulsan College of Medicine, 88 Olympic-ro 43-gil, Songpa-gu, Seoul, 05505 South Korea; 3 Department of Stem Cell Biology, School of Medicine, Konkuk University, Seoul, South Korea; 4 Mirae Cell Bio Co. Ltd., Seoul, South Korea

**Keywords:** Detrusor underactivity, Chronic bladder ischemia, Multipotent mesenchymal stem cells, Embryonic stem cells

## Abstract

**Background:**

The therapeutic effects of human embryonic stem cell-derived multipotent mesenchymal stem cells (M-MSCs) were evaluated for detrusor underactivity (DUA) in a rat model with atherosclerosis-induced chronic bladder ischemia (CBI) and associated mechanisms.

**Methods:**

Sixteen-week-old male Sprague–Dawley rats were divided into five groups (*n* = 10). The DUA groups underwent 30 bilateral repetitions of endothelial injury to the iliac arteries to induce CBI, while the sham control group underwent a sham operation. All rats used in this study received a 1.25% cholesterol diet for 8 weeks. M-MSCs at a density of 2.5, 5.0, or 10.0 × 10^5^ cells (250 K, 500 K, or 1000 K; K = a thousand) were injected directly into the bladder 7 weeks post-injury, while the sham and DUA group were treated only with vehicle (phosphate buffer solution). One week after M-MSC injection, awake cystometry was performed on the rats. Then, the bladders were harvested, studied in an organ bath, and prepared for histological and gene expression analyses.

**Results:**

CBI by iliac artery injury reproduced voiding defects characteristic of DUA with decreased micturition pressure, increased micturition interval, and a larger residual volume. The pathological DUA properties were improved by M-MSC treatment in a dose-dependent manner, with the 1000 K group producing the best efficacy. Histological analysis revealed that M-MSC therapy reduced CBI-induced injuries including bladder fibrosis, muscular loss, and apoptosis. Transplanted M-MSCs mainly engrafted as vimentin and NG2 positive pericytes rather than myocytes, leading to increased angiogenesis in the CBI bladder. Transcriptomes of the CBI-injured bladders were characterized by the complement system, inflammatory, and ion transport-related pathways, which were restored by M-MSC therapy.

**Conclusions:**

Single injection of M-MSCs directly into the bladder of a CBI-induced DUA rat model improved voiding profiles and repaired the bladder muscle atrophy in a dose-dependent manner.

**Graphical abstract:**

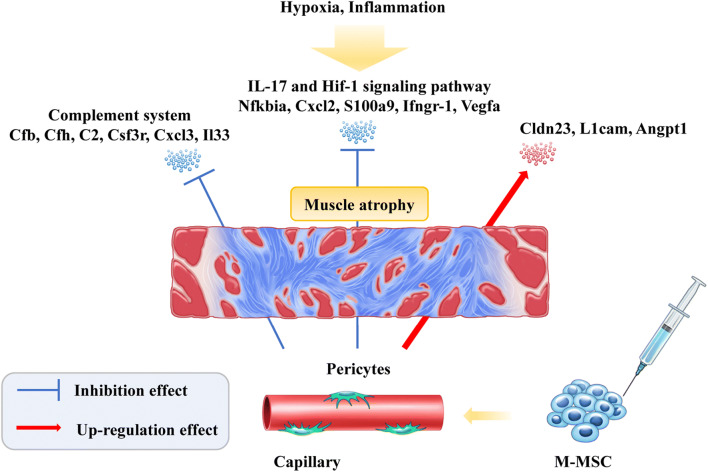

**Supplementary Information:**

The online version contains supplementary material available at 10.1007/s12015-021-10204-z.

## Introduction

Detrusor underactivity (DUA) is defined as “low detrusor pressure or short detrusor contraction time, usually in combination with a low urine flow rate resulting in prolonged bladder emptying and/or failure to achieve complete bladder emptying within a normal time span” by the international continence society standardization [1, 2]. To date, the available therapeutic options for DUA are suboptimal, with the majority of patients suffering from persistent discomfort, severely deteriorated quality of life, and complications, including recurrent urinary tract infections and urinary retention [3]. Research to develop more effective, long-term therapies for DUA with improved patient outcomes is ongoing.

Stem cell therapy is emerging as a potential treatment option for a wide range of intractable diseases including bladder voiding dysfunction disorders [4, 5]. Stem cells are characterized as cells that can self-renew and possess differentiation potency, which can be capitalized on to replace the damaged cells. Among the different types of stem cells, mesenchymal stem cells (MSCs) are considered as a reliable source for stem cell therapy. Besides their regenerative capacity, the MSCs can migrate to the damaged tissues and exhibit paracrine effects, such as recruitment of endogenous progenitor cells and secretion of growth-factors that can favor tissue regeneration. MSCs can been isolated from adult or fetal tissues [5], or they can be also derived from pluripotent stem cells (PSCs) such as embryonic stem cells (ESCs) and induced PSCs (iPSCs) [6–8]. The beneficial effects of MSC therapy include the generation of various preclinical bladder dysfunction models and reports of positive patient outcomes from clinical trials [9–17].

Previously, we investigated the therapeutic effects of multipotent MSCs (M-MSCs) derived from human ESCs in a streptozotocin-induced diabetic rat model of DUA. The transplanted M-MSCs integrated into pericytes, which provided favorable micro-environments for detrusor muscle regeneration [12]. In the present study, we aimed to investigate the therapeutic effects of M-MSC treatment in a DUA rat model with extensive vascular endothelial damage of the iliac arteries. Further, we aimed to investigate the underlying pathophysiological mechanisms of DUA for targeted therapeutics.

## Methods

### Ethics Statement and Study Approval

Animal experiments were approved by the Institutional Animal Care and Use Committee of the University of Ulsan College of Medicine (IACUC-2019-12-004) and performed in accordance with the guidelines and regulations.

### Study Design

Sixteen-week-old male Sprague–Dawley rats were divided into five groups: sham (*n* = 9), DUA (n = 9), DUA + 250 K M-MSCs (*n* = 10), DUA + 500 K M-MSCs (n = 9), and DUA + 1000 K M-MSCs (n = 10) (K = a thousand). All animals were anesthetized by intraperitoneal injection of 30 mg/kg Zoletil (Virbac Laboratories, Carros, France) prior to operation. Rats in the DUA groups had a 2-French Fogarty arterial embolectomy catheter (E-060-2F; Edwards Lifesciences, Irvine, CA, USA) inserted into the common iliac artery via the femoral artery. The balloon was passed from the common iliac artery to the femoral artery over 30 repetitions in the inflated state, and then the same procedure was repeated bilaterally. The sham group underwent sham operation. All rats received a 1.25% cholesterol diet (D12336; Research Diets, New Brunswick, NJ, USA) for 8 weeks. Seven weeks after the operation, the DUA plus stem cell treatment groups were injected with 2.5, 5.0, or 10.0 × 10^5^ M-MSCs (250 K, 500 K, or 1000 K, respectively) directly at the serosa of the anterior bladder wall. For the sham and DUA groups, phosphate-buffer solution (PBS) was injected.

Animals that did not survive in catheter implantation or bladder manipulation were excluded from subsequent analyses. Additional rats (*n* = 6) were used for the organ bath study. The M-MSCs used in the stem cell treatments were maintained up to seven passages only to ensure their functionality as previously described [9, 10, 18].

### Evaluation of Bladder Voiding Function

One week after M-MSC injection, the experimental groups were evaluated by awake cystometry. Bladder voiding function was evaluated in unrestrained, awake-state rats in metabolic cages as previously described [9, 10, 12]. Detrusor pressure was defined as [intravesical pressure (IVP) – intra-abdominal pressure (IAP)]. The contractility of the bladder tissues was measured by the organ bath study, as previously described [12, 19]. In brief, longitudinal strips of posterior bladder wall were normalized to weight per 1 g and then mounted in 5 mL organ baths containing Krebs solution (118 mM NaCl, 5.0 mM KCl, 2.5 mM CaCl_2_, 1.0 mM MgSO_4_, 30 mM NaHCO_3_, 1.0 mM KH2PO_4_, and 11.4 mM glucose, pH 7.4), and maintained at 37 °C with 5% CO_2_ and 95% O_2_ continuously supplied. The contractile response to 80 mM KCl (P9333; Sigma-Aldrich, St. Louis, MO, USA), EFS (electrical field stimulation, 1, 2, 4, 8, 16, and 32 Hz), 1 mM ATP (A2383; Sigma-Aldrich), or carbachol concentration (PHR1511; Sigma-Aldrich; 1 nM to 1 mM) were recorded as previously described [12, 19]. An electrical pulse (1 millisecond pulse width and 80 V in bath) was delivered using a stimulator (D-7806; Hugo Sachs Elektronik, Germany) for 5 s at increasing frequencies (1, 2, 4, 8, 16, and 32 Hz), with 5 min intervals between electrical field stimulations.

### Histological and Immunostaining Analysis

Next, the bladder tissues were harvested for histological and gene expression analyses. For histological analysis, collagen deposition, bladder muscle atrophy, and angiogenesis were assessed with Masson’s trichrome staining (Junsei Chemical, Tokyo, Japan) and immunohistochemistry with anti-α-smooth muscle actin (α-SMA) (ab7817; Abcam, Cambridge, UK), and anti-CD31 (sc-376,764; Santa Cruz Biotechnology, Dallas, TX, USA) antibody staining, respectively. Apoptosis in each layer of bladder (urothelium and muscle layer) was assessed by terminal deoxynucleotidyl transferase dUTP nick-end labeling staining (1,684,795; TUNEL, Roche, Mannheim, Germany), and the nuclei were counterstained with 4′6-diamnio-2-phenylindole (D9542; Sigma-Aldrich). Two representative areas were selected at random from each slide and used for quantification analysis using Image Pro 5.0 software (Media-Cybernetics, Rockville, MD, USA).

The distribution and cellular properties of transplanted M-MSCs were evaluated by immunohistochemical analysis of the bladders with human β2-microglobulin (hB2MG) (SC80668; Santa Cruz Biotechnology, Dallas, TX, USA) and by co-staining with antibodies specific for vimentin (#5741; Cell Signaling Technology, Danvers, MA, USA), α-SMA (ab5694; Abcam), NG2 (ab129051; Abcam) and CD31 (ab28364; Abcam). These proteins were visualized by Alexa Fluor 488-conjugated (A11001) or Alexa Fluor 564-conjugated (A11010) anti-mouse or anti-rabbit secondary antibodies (Molecular Probes, Grand Island, NY, USA). Images were acquired using a ZEISS LSM800 confocal microscope system (Carl Zeiss, Munich, Germany).

### Transcriptome and Gene Expression Analysis

Publicly available transcriptome datasets (GEO series accession number: GSE122060) [19] were used to compare gene expression data from CBI and sham-operated rat bladders. Differentially expressed genes were determined as 1.5 fold up- or down-regulation, with *p* < 0.05 defined as the cutoff values. Transcriptomic features were analyzed using MetaCore (Clarivate Analytics, Philadelphia, PA, USA) with default settings, which provided gene networks, biofunctions, and canonical pathways for representing CBI bladders. Details of the statistical values and significant genes for each analysis are described in the **Supplementary Dataset** **1**. The significance of each candidate gene was individually validated by real-time quantitative PCR (RQ-PCR) analysis, as previously described [20, 21]. The sequences of primers used in this study were described in the **Supplementary Dataset** **2**.

### Statistical Analysis

Data are reported as the mean standard error of the mean (SEM) and were analyzed using GraphPad Prism 7.0 software (GraphPad Software, La Jolla, CA, USA). Differences and significance were verified by one- or two-way ANOVA followed by Bonferroni post-hoc tests. A *p* value <0.05 was considered as statistically significant.

## Results

### Bladder Function Evaluation

**Fig. 1:**
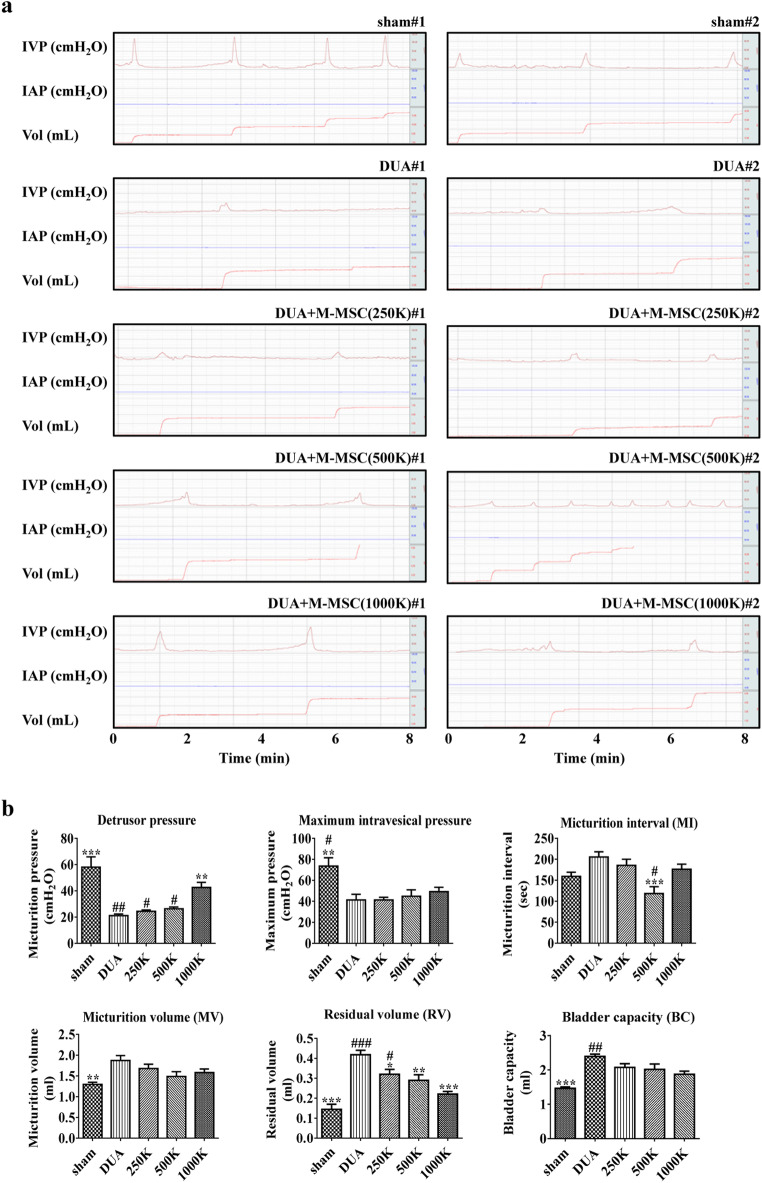
**M-MSC injection restored bladder function in DUA rat models.** (**a**) Representative awake cystometry results and (**b**) quantitative analysis of bladder voiding parameters 1 week post-injection of either 0.25 ×, 0.5 ×, or 1.0 × 10^6^ M-MSCs (250 K, 500 K, or 1000 K groups, K = a thousand) into rat bladders. All quantitative data are presented as mean ± SEM. *p < 0.05, **p < 0.01, ***p < 0.001 compared with the DUA group; #p < 0.05, ##p < 0.01, ###p < 0.001 compared with the 1000 K group. One-way ANOVA with Bonferroni post-hoc test was used for statistical analysis. IVP, intravesical pressure; IAP, intra-abdominal pressure

Previously, we reported that DUA could be induced in a rat model of CBI with 30 bilateral repetitions of iliac arterial injury, followed by a 1.25% high cholesterol diet for 8 weeks [19]. In the present study, we investigated whether M-MSCs derived from human ESCs can show the therapeutic potency for treating DUA in this rat model. To address this issue, we transplanted three different cell dosages (0.25, 0.5, and 1.0 × 10^6^ cells; denoted 250 K, 500 K, and 1000 K, respectively) of M-MSCs directly into the bladders at 7 weeks post-CBI injury. As a control, we administered PBS to the CBI-induced DUA rat model (DUA group) and the sham-operated rats (Sham group). In line with our previous report [19], the DUA group presented with decreased detrusor pressure and larger micturition volume, post-void residual, and bladder capacity than the sham group (Fig. 1a and b). A single administration of M-MSCs restored detrusor pressure in a dose-dependent manner; however, the beneficial effects of M-MSC therapy were suboptimal for restoring micturition interval and volume. Conversely, injection of 1000 K M-MSCs significantly reduced the residual volume, resulting in decreased bladder capacity, compared with the DUA group (Fig. 1b).

### Organ Bath Study

**Fig. 2:**
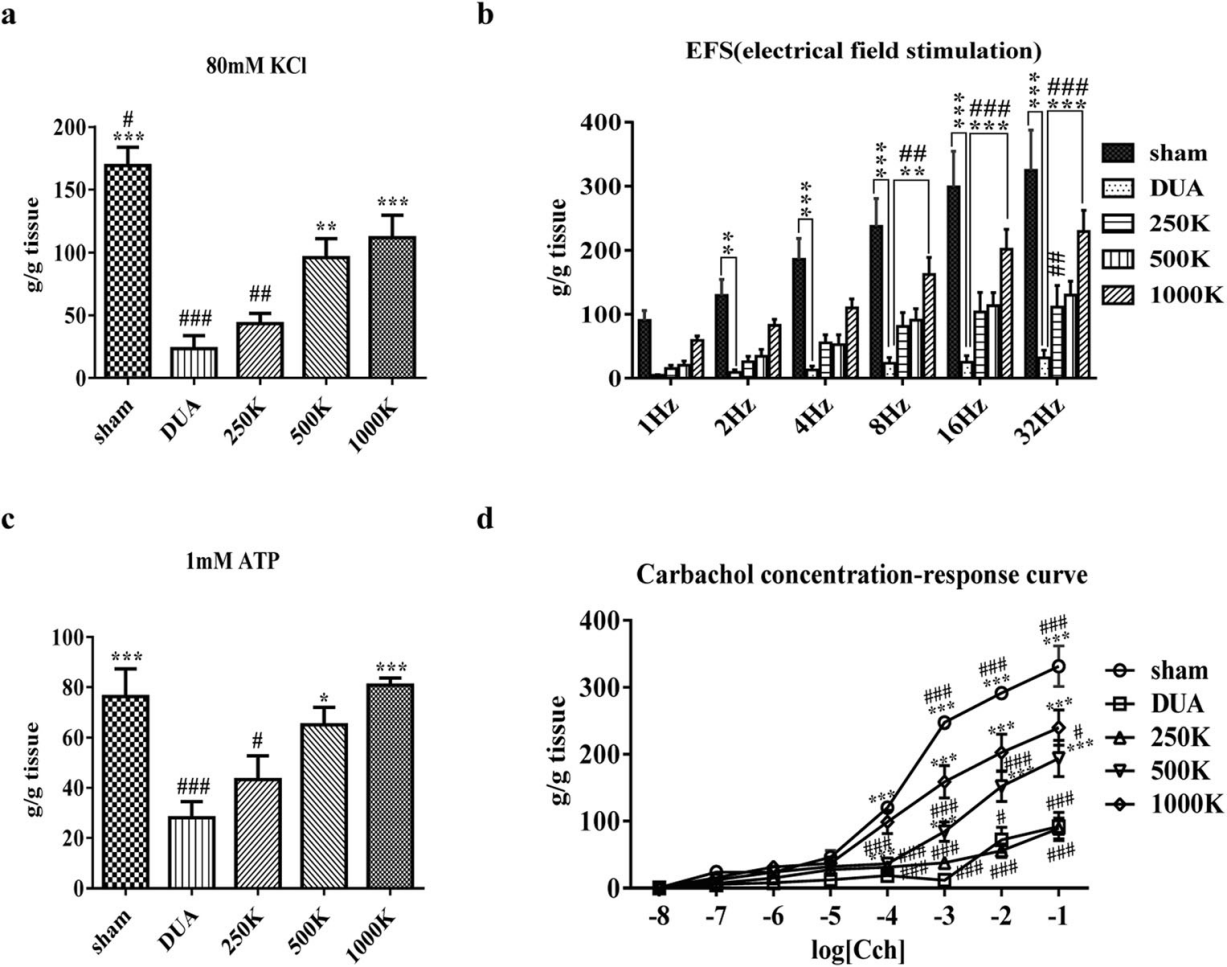
**Repair of bladder contractility by M-MSC therapy.** (**a-d**) Organ bath analysis for evaluating the contractile response of bladder muscle strips to either 80 mM KCl (**a**), electrical field stimulation (**b**), 1 mM ATP **(c)**, or carbachol, as indicated (**d**). All quantification data are presented as mean ± SEM (12 bladder strips from six independent rats). *p < 0.05, **p < 0.01, ***p < 0.001 compared with the DUA group; #p < 0.05, ##*p* < 0.01, ###*p* < 0.001 compared with the 1000 K group with one- (**a and c**) or two- (**b** and **d**) ANOVA with Bonferroni post-tests

Bladder strips from the DUA group overall demonstrated significantly deteriorated contractile responses to various stimuli including 80 mM KCl, electrical field stimulation, 1 mM ATP, and carbachol concentration response, compared with the sham group (Fig. 2a-d). In line with the awake cystometry results, M-MSC injection restored these contractility defects in a dose-dependent manner, with 1000 K M-MSC treatment exhibiting the highest potency in all the stimulations. Taken together, these results demonstrate that a single local administration of 1.0 × 10^6^ M-MSCs was more effective at restoring bladder voiding function and contractibility induced by a high degree of atherosclerotic occlusion.

### Histological Analysis

**Fig. 3:**
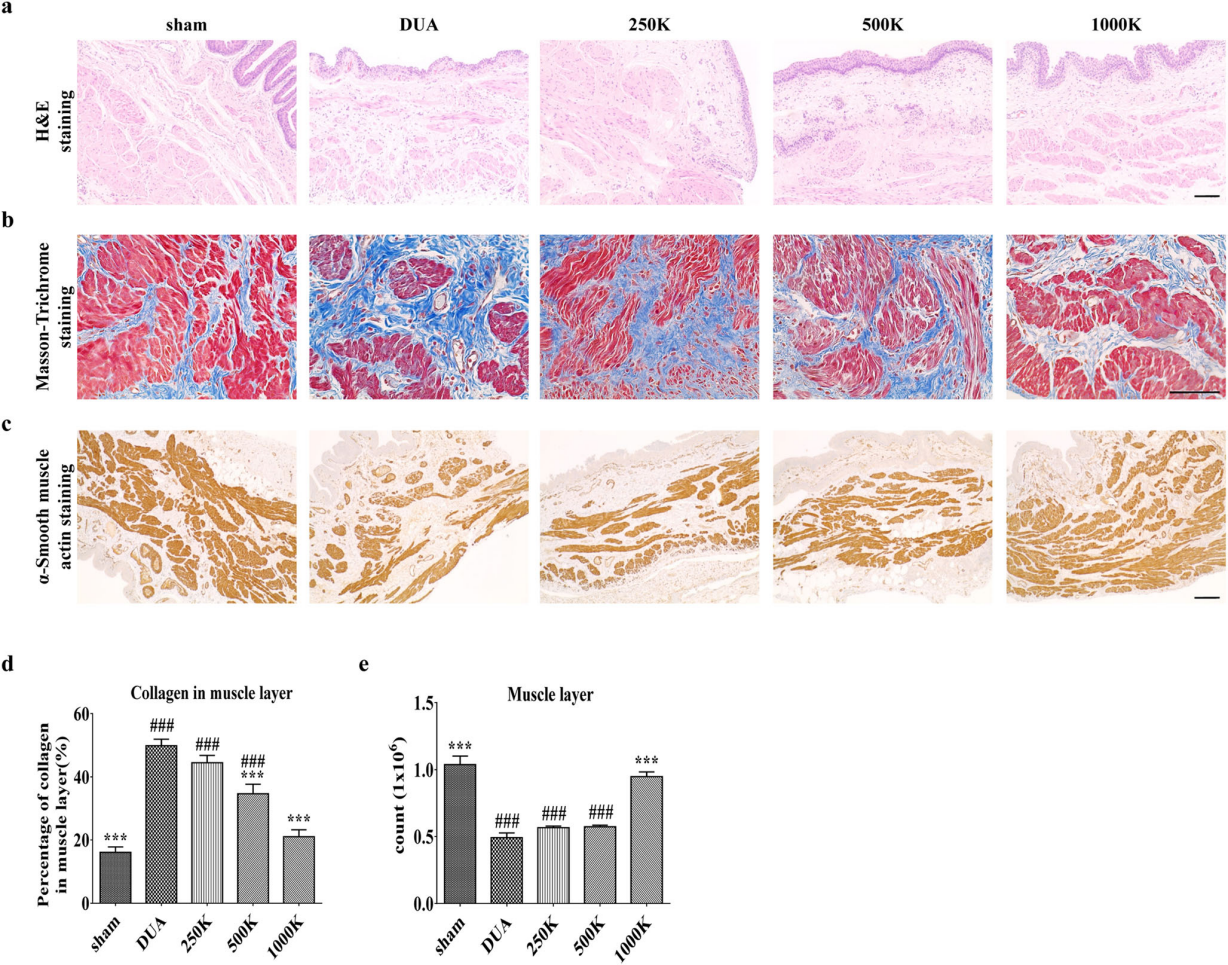
**M-MSCs injection repaired histological injury in CBI bladders. (a and b)** Representative images for hematoxylin and eosin (H&E; magnification ×100, scale bar = 200 μm, **A**) and Masson’s trichrome (magnification ×400, scale bar = 200 μm, **b**) staining in the bladder tissues 1 week after transplantation of the indicated dosage M-MSCs. The tissue fibrosis was stained in blue. **(c)** Representative images for immuno-histochemical staining of α-smooth muscle actin (α-SMA, magnification ×100, scale bar = 200 μm) in the indicated bladders. Nuclei were stained with Mayer’s hematoxylin. **(d and e)** Quantification of histological examinations for fibrosis **(d)** and α-SMA stained muscle fiber **(e)**. All quantitative data are presented as the mean ± SEM (*n* = 9 or 10). *p < 0.05, **p < 0.01, ***p < 0.001 compared with the DUA group; #p < 0.05, ##p < 0.001, ###p < 0.001 compared with the 1000 K group with one-way ANOVA with Bonferroni post-tests

**Fig. 4:**
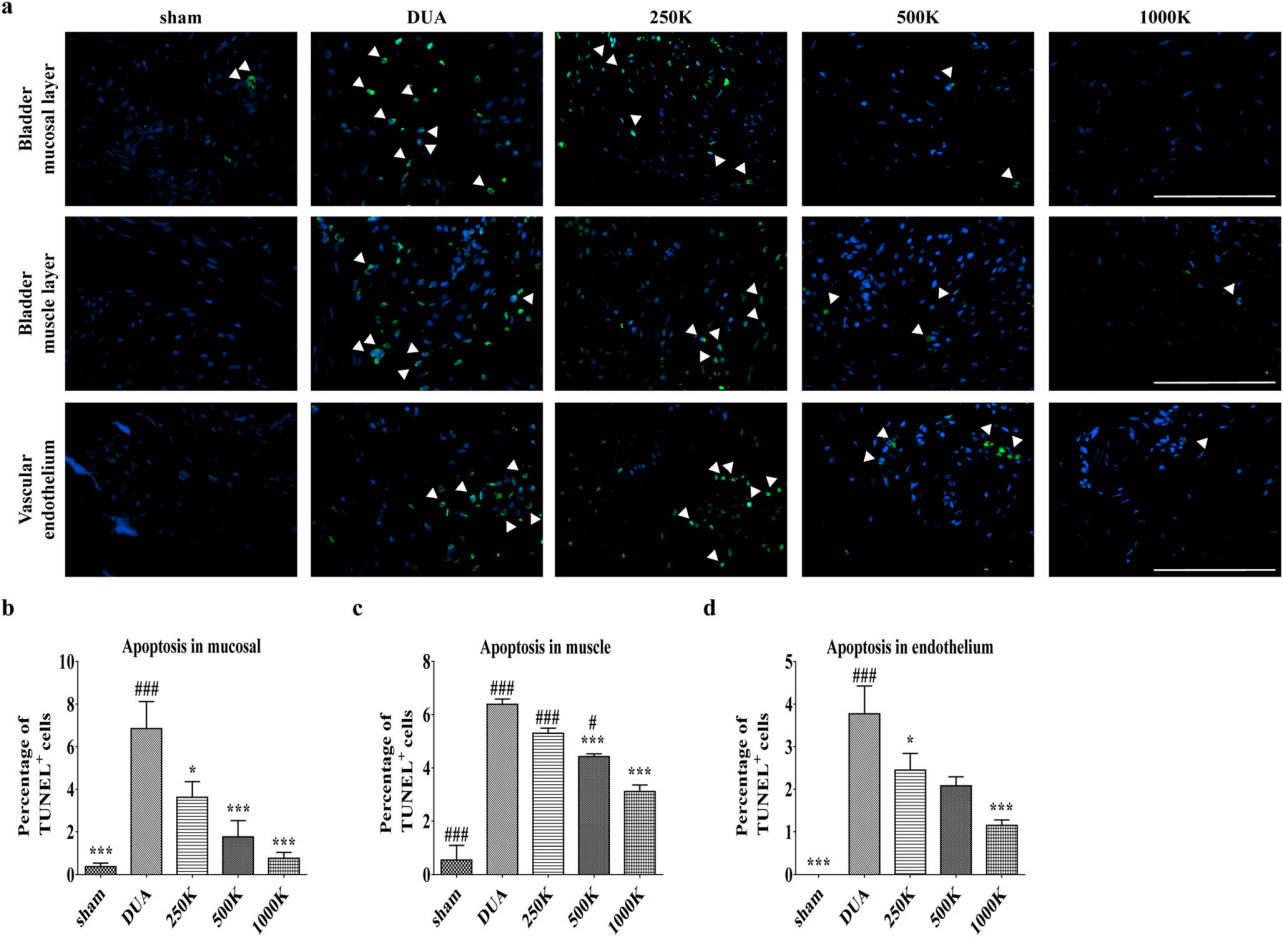
**M-MSC therapy protected the apoptotic response in CBI bladders. (a)** Representative images of TUNEL staining for apoptotic cells (green, magnification ×400, scale bar = 200 μm) in the mucosal and muscle layers, and vascular endothelium of bladders in the indicated groups. Nuclei were stained with DAPI. **(b-d)** Quantification of percentage of apoptotic cells (arrowhead in **a**) by calculating the ratio of apoptotic cells (TUNEL positive) to total cells (DAPI-stained nuclei). All quantitative data are presented as the mean ± SEM (n = 9 or 10). *p < 0.05, **p < 0.01, ***p < 0.001 compared with the DUA group; #p < 0.05, ##p < 0.001, ###p < 0.001 compared with the 1000 K group with one-way ANOVA with Bonferroni post-tests

We next examined whether M-MSC therapy can regenerate the histological damage characteristic of DUA bladders. Rat bladders in the DUA group presented with atrophy of bladder muscle (Fig. 3a) and accompanied fibrosis indicated by accumulation of collagen fibers based on strong Masson’s trichrome staining (Fig. 3b). The muscular degeneration was confirmed by immunohistochemical staining of α-SMA protein (Fig. 3c). Consistent with these histological injuries, the DUA bladders showed a significant increase of apoptosis in both the urothelial and muscular layer of the bladder along with endothelium of bladder vasculature (Fig. 4a). Of importance, M-MSC injection into the rats alleviated the characteristic DUA histological injuries including fibrosis, muscular degeneration, and increased apoptosis of the bladder muscle tissue (Figs. 3 and 4). Rat bladders treated with 1000 K M-MSCs had a significant improvement of tissue fibrosis and muscle atrophy, and reduced apoptosis of muscle fibers, compared with the 250 K and 500 K MSC treatment groups (Fig. 3e and d**;** Fig. 4b-d). These results indicate a dose-dependent therapeutic effect of M-MSC therapy for DUA.

### Characterization of Cellular Properties of the Engrafted M-MSCs

**Fig. 5:**
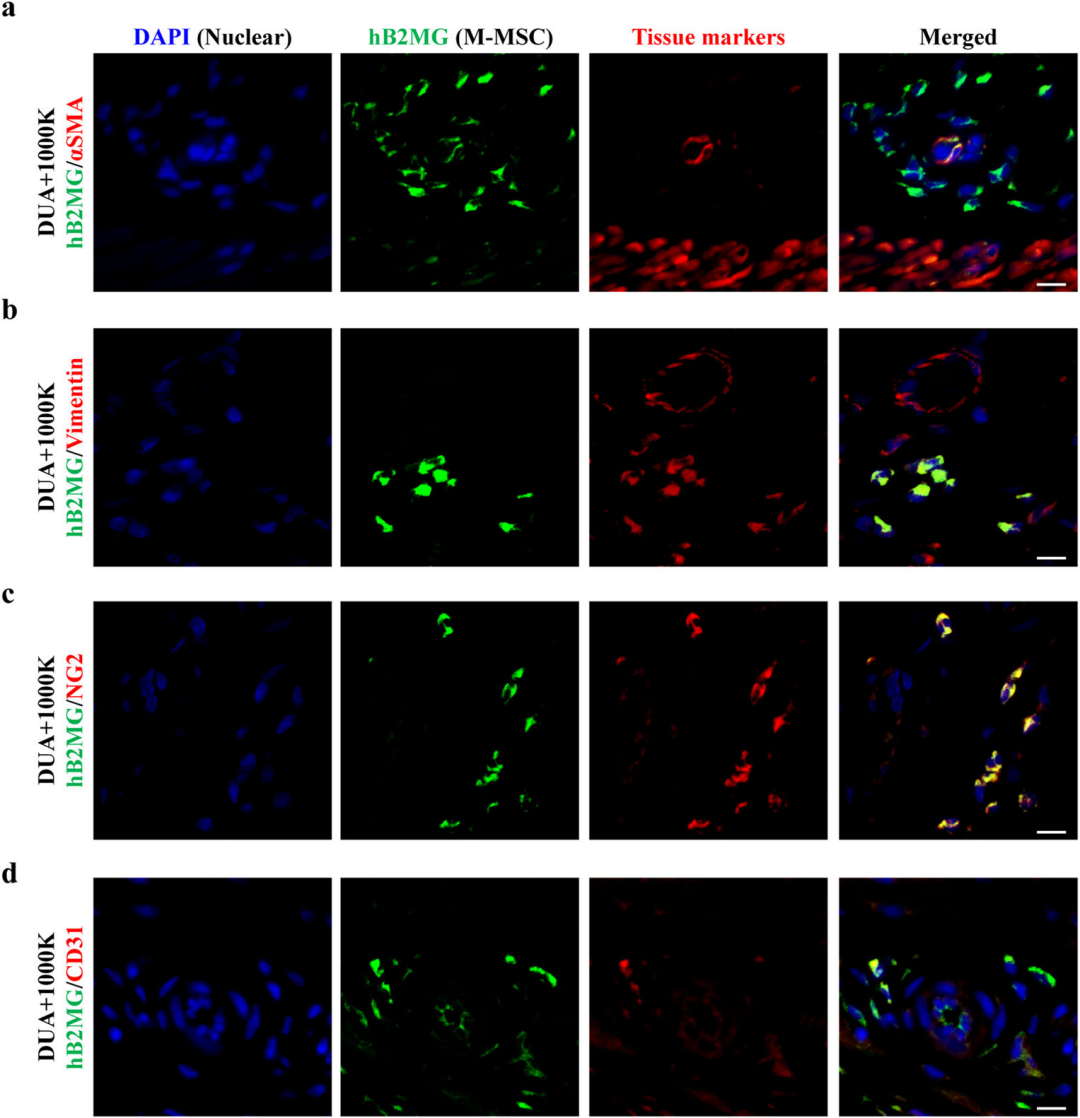
**Cellular lineages of transplanted M-MSCs in the CBI bladders. (a-c)** Representative confocal microscopic images (magnification ×1000, scale bar = 10 μm) of human B2MG (hB2MG, green) co-stained with either α-SMA myocyte **(a)**, vimentin stromal cell **(b)**, NG2 pericyte **(c),** or CD31 endothelial cell **(d)** markers in bladder sections harvested from DUA + 1000 K rats at 1 week post-transplant. Nuclei were stained with DAPI (blue). Note that M-MSCs mainly engrafted as pericytes, not endothelial cells in vessels near muscle fibers in the CBI bladders

Next, to determine the in vivo distribution and cellular differentiation lineage of the transplanted M-MSCs, the engrafted M-MSCs were detected by immunostaining with the human antigen hB2MG, and co-stained with either α-SMA, a muscle marker; vimentin, a mesenchymal marker; or NG2, a pericyte maker. The majority of hB2MG^+^ engrafted cells were localized between muscle and serosa of the bladder at the site of M-MSCs injection. In particular, the engrafted hB2MG positive (^+^) cells were frequently observed near but not in the muscle fibers, and they expressed minimal α-SMA protein (Fig. 5a), suggesting that the transplanted M-MSCs may not contribute toward the myocyte in the CBI-injured bladders. Instead, the hB2MG^+^ cells were localized in close proximity to bladder vessels and co-stained with the NG2 pericyte and vimentin stromal marker proteins (Fig. 5b and c). The results indicated that M-MSCs mainly engrafted as pericytes to support paracrine effects for repairing tissue damage in the CBI bladders.

### The Role of M-MSC Therapy in Angiogenesis of the CBI Bladders

**Fig. 6:**
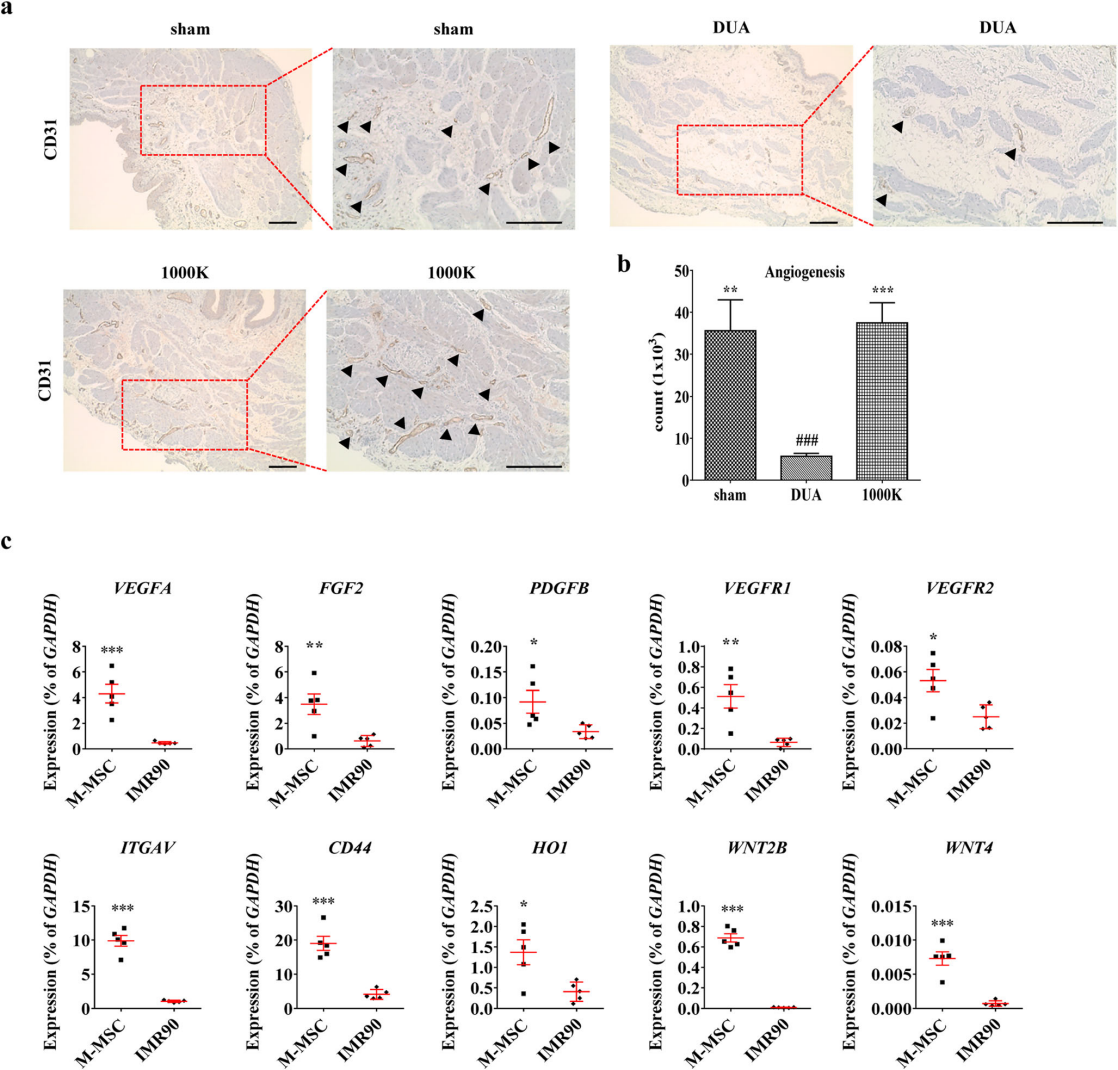
**M-MSCs injection stimulated angiogenesis in CBI bladders. (a)** Representative images of immuno-histochemical staining of CD31 (magnification ×100, scale bar = 200 μm) in the bladder tissues 1 week after transplantation of the indicated dosage of M-MSCs. Photomicrographs with higher magnification (magnification ×200, scale bar = 200 μm) are shown in the right corner of each panel. The arrowheads indicate the CD31 positive (CD31^+^) vessels. The nuclei were stained with Mayer’s hematoxylin. **(b)** Quantification of the CD31^+^ vessels in the indicated bladders. Quantitative data are presented as the mean ± SEM (n = 9 or 10). **p < 0.01, ***p < 0.001 compared with the DUA group; ###p < 0.001 compared with the 1000 K group with one-way ANOVA with Bonferroni post-tests. **(c)** Expression of transcripts of genes related to the angiogenesis and WNT signaling pathways in the M-MSCs and IMR90, a human primary lung fibroblast. Expression is presented as % *GAPDH* and shown as a dot plot of mean ± SEM (*n* = 5). *p < 0.05, **p < 0.01, ***p < 0.001 compared by the non-parametric Mann–Whitney *U* test

Induction of angiogenesis is an important mechanism for elucidating the beneficial outcome of MSC therapy [22]. To address this issue, we quantified the blood vessel content in the rat bladders by immunohistochemical analysis with CD31, an endothelial cell marker. In line with the engraftment of M-MSCs as a pericyte (Fig. 5b and c), the content of CD31^+^ vessels in the CBI-injured bladders was increased by the administration of M-MSCs (Fig. 6a and b). However, the majority of hB2MG^+^ cells expressed practically no CD31 antigen (Fig. 5d), indicating that pericytes near blood vessels, not endothelial cells, were the major cellular fate of the engrafted M-MSCs in the CBI bladders.

To obtain more mechanistic insights, we examined the expression level of several factors related to angiogenesis and the repair of the bladder injury [4, 23]. The gene expression analysis revealed that M-MSCs, compared with IMR90, a differentially normal fibroblast cell, had significantly increased expression of a subset of pro-angiogenic factors and their cognate receptors, including vascular endothelial growth factor-A (*VEGFA*), platelet-derived growth factors (*PDGF-A, -B, and -D*), fibroblast growth factor-2 (*FGF2*), transforming growth factor β-1 (*TGFB1*), VEGF receptor-1 (*VEGFR1*), integrin subunit α-V (*ITGAV*), angiopoietin-1 receptor (*TEK*), and *CD44* (Fig. 6c**and** Suppl Fig. 1). In addition, M-MSCs up-regulated their expression of WNT family member genes (e.g., *WNT2*, *WNT4*, and *WNT5B*), which play an important role in repair of the bladder injury [9, 10, 14, 24]. Taken together, these results support the finding that M-MSCs, mainly engrafted as pericytes in blood vessels near bladder muscle fibers, stimulate angiogenesis in the CBI injured bladder, which might be crucial for their therapeutic potency.

### Gene Expression Analysis

**Fig. 7:**
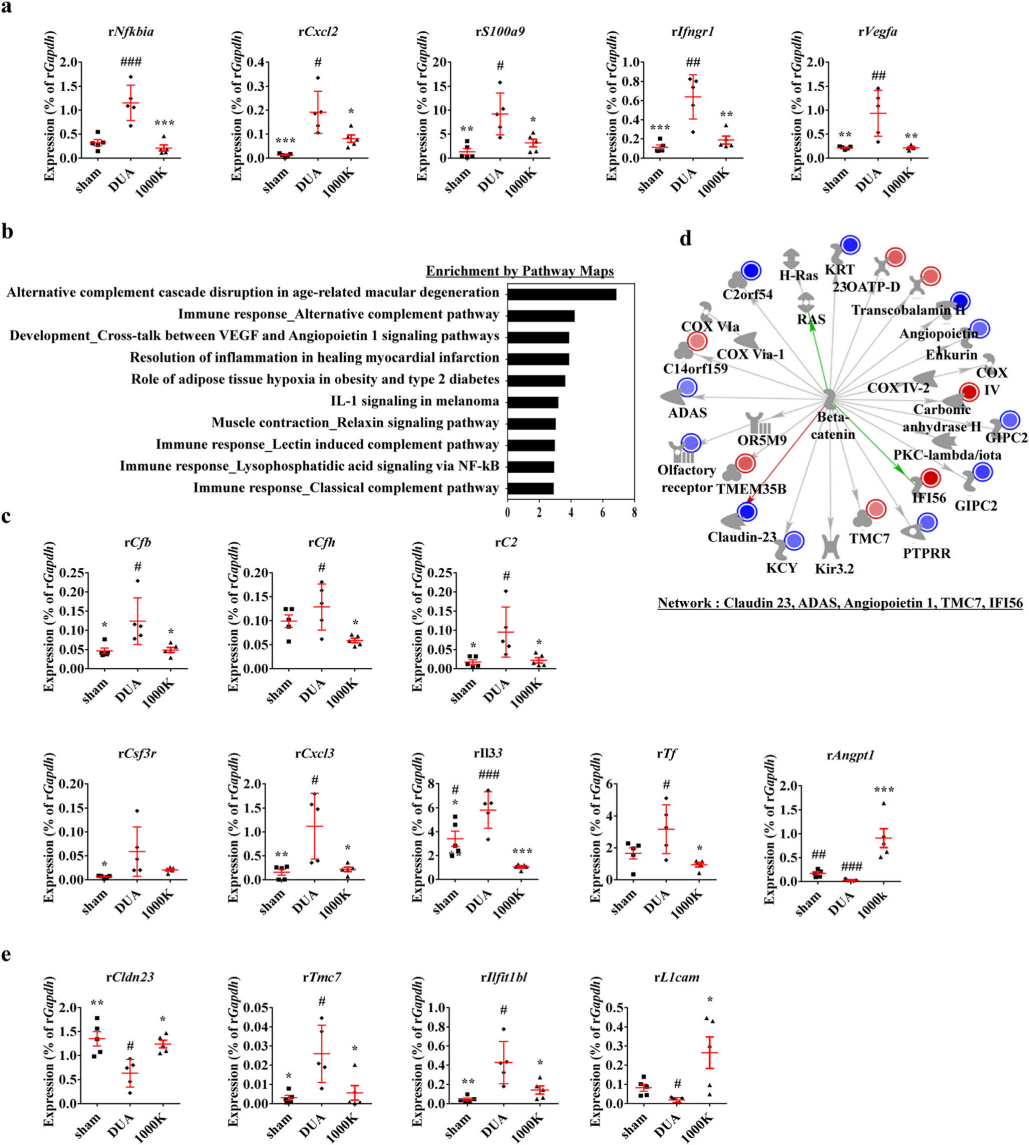
**Gene expression change in CBI-induced DUA bladders following M-MSC therapy. (a)** Real-time qPCR analysis of genes relating to the IL-17 and HIF-1 signaling pathways in the indicated bladders. **(b and d)** MetaCore analysis of DUA and sham bladders revealed the top ten pathway maps **(b)** and a representative gene network associated with the sensory perception of chemical stimuli **(d)**. The gene network is illustrated by overlaying experimental values as fold changes in CBI versus sham samples. Up- and down-regulated genes are indicated in red and green, respectively. **(c and e)** Real-time qPCR analysis of genes related to the complement system, inflammatory, and ion transport-related pathways **(d),** as well as genes involved in networks characteristic of CBI **(e)** in the indicated bladders. Expression is presented as % of *Gapdh* expression and shown as a dot plot of mean SEM (n = 5). *p < 0.05, **p < 0.01, ***p < 0.001 compared with the DUA group; #p < 0.05, ##p < 0.01, ###p < 0.001 compared with the 1000 K group with one-way ANOVA with Bonferroni post-tests

A previous transcriptomic study reported that CBI-induced bladder injury characteristically up-regulated the genes involved in the IL-17 and HIF-1 signaling pathways [19]. For example, the expression of NFKB inhibitor-alpha (*Nfkbia*), C-X-C motif chemokine ligand-2 (*Cxcl-2*), and S100 calcium binding protein-A9 (*S100a9*) representing the IL-17 pathway was significantly increased in the CBI-injured bladders. In addition, the expression of interferon gamma receptor-1 (*Ifngr-1*) and *Vegfa* were up-regulated as HIF-1 pathways. Of importance, M-MSC therapy significantly prevented the dysfunction of these IL-17- or HIF-1-related genes (Fig. 7a).

To identify the driving genes underlying DUA pathogenesis induced by ischemic vascular injury, we further analyzed the published transcriptome datasets for sham and DUA bladders [19] using the MetaCore transcriptome analysis tool that provides the gene networks, biological processes, and pathway maps. Compared with the sham group, CBI bladders exhibited characteristic changes in the complement immune response-, inflammation-, and ion transport-related pathways (Fig. 7b**and** Suppl Fig. 2a-2c). The molecular features of CBI bladders were elucidated by examining the altered expression of complement factor-B or -H (*Cfb* and *Cfh*), complement C2 (*C2*), Angiopoietin 1 (*Angpt-1*), colony stimulating factor 3 receptor (*Csf3r*), C-X-C motif chemokine ligand-3 (*Cxcl-3*) genes, interleukin-33 (*Il-33*), and transferrin (*Tf*) (Fig. 7c. The transplantation of M-MSCs significantly restored the altered expression of *Cfh* and *Tf* as well as the majority of the inflammatory genes in the bladders.

Furthermore, MetaCore gene network analysis revealed that the CBI bladders were characterized by the VEGF-A and I-κB associated gene networks involving *Vegf-a*, *Ikb*, and the L1 cell adhesion molecule (*L1cam*) **(**Suppl. Fig. 2d**)**, as well as networks related to the sensory perception of chemical stimuli, including claudin-23 (*Cldn23*), alkylglycerone phosphate synthase (*Agps*, also known as *Adas)*, *Angpt-1*, transmembrane Channel Like 7 (*Tmc7*), and interferon-induced protein with tetratricopeptide repeats-1 (*Ifit-1*, also known as *Ifi-56*) (Fig. 7d). Accordingly, CBI injury significantly down-regulated *Cldn23* and up-regulated *Tmc7* in the bladders treated with M-MSCs (Fig. 7e). Collectively, these results demonstrate the novel significance of the complement system and ion transport-related pathways. Further, these results indicate that CBI-induced DUA pathogenesis leads to an NF-κB- and VEGF-A-mediated inflammatory response, which may be important when considering the applications of M-MSC therapy.

## Discussion

In this preclinical study, we demonstrate the therapeutic effects of M-MSC transplantation in a CBI-induced rat model of DUA. Following transplantation, the injected M-MSCs mainly integrated into pericytes nearby muscle fibers of the rat bladders and exhibited paracrine effects to repair vascular insult and detrusor muscle damage. Mechanistically, M-MSC therapy prevented the NF-κB-mediated inflammatory and complement system response in the CBI-induced bladders.

In human DUA patients, a common symptom is an underactive bladder that is characterized by a slow urine stream, hesitancy, and straining to void, with or without a feeling of incomplete bladder emptying [25]. Diagnosis of underactive bladder is frustrating as current therapeutic options have suboptimal and limited efficacy. In the early phase of DUA, patients are able to void with abdominal straining and oral medication but might complain of a weak stream and residual urine sensation. However, as DUA persists, progressive bladder remodeling results in a decompensated state with urothelial dysfunction, neuron and smooth muscle degeneration, and high levels of extracellular matrix deposition in the bladder [26]. Timely intervention to prevent irreversible changes of detrusor is necessary.

The current treatment strategies for DUA aim to improve detrusor contractility, reducing bladder outlet resistance or direct drainage of urine. Surgical intervention to reduce bladder outlet resistance and direct drainage of urine is relatively invasive compared with medical therapy. Direct drainage of urine is not a definite resolution for DUA as it only circumvents the existing problem [27]. In addition, effective bladder emptying cannot be achieved if adequate detrusor contraction is absent. Considering that normal voiding is achieved by adequate, continuous detrusor contraction, which leads to complete bladder emptying within a normal time span, future investigations to fulfill the unmet needs of DUA should focus on restoring the detrusor contractility before the golden time.

Stem cell therapy seems to be an adequate candidate for restoring detrusor contractility [4, 12]. Contemporary concepts in the pathogenesis of DUA are complex, such that its etiological factors can be classified as idiopathic, neurogenic, myogenic, iatrogenic, and functional [28]. Our CBI DUA model presented with myogenic degeneration, increased apoptosis in both the urothelial and muscular layers of the bladder, and increased collagen deposition. A single administration of M-MSCs successfully alleviated both the histological and functional abnormalities by reversing muscle atrophy and reducing the inflammatory and complement system response in CBI-induced rat bladders. Therefore, the present study provides an in vivo proof of concept that MSC therapy is a viable option for treating DUA.

The application of autologous muscle-derived mesenchymal stem cells (AMDC) has been reported in several clinical trials to treat stress urinary incontinence patients. Recently, Gilleran et al., reported the first regulatory approved clinical trial, which evaluated the safety and efficacy of intradetrusor injected AMDCs in 20 non-neurogenic DUA patients [29]. The study subjects received approximately 30 transurethral injections of 0.5 mL delivered to the bladder (125 million AMDC/15 mL). The initial end point was post-injection 6-months; however, all participants asked for a second injection due to satisfactory results so the follow-up assessments were made at post-injection 1, 3, 6, and 12 months. At the primary outcome point of 12 months, 11 out of 19 patients (58%) reported a global response assessment ≥5, showing slight to marked improvement in their symptoms. In addition, improvement of voiding efficiency was observed in many subjects who were catheter dependent at baseline. No AMDC-related serious adverse events were reported. The reported adverse events included injection related and biopsy related complications. The main differences between this clinical trial and our preclinical study are the injection route and the type of stem cells used. As there are no currently available devices, such as a cystoscope with an injection channel for rats, the M-MSCs were directly injected into the rat bladder. However, if our study is clinically applied, M-MSCs will be injected via a transurethral route as it is familiar to urologists and is minimally invasive.

Large-scale production of MSCs from adult tissues adversely resulted in the loss of their primitive functions. In addition, adult-tissue derived MSCs, especially those from aged donors, have limited proliferative capacity in vitro due to replicative senescence [30–33], and MSCs at high passage number are more likely to trigger an innate immune attack upon transplantation [34]. To overcome these issues, M-MSCs used in this study were derived from hESCs, which have been suggested as a cost-effective alternative source due to their pluripotency and unlimited expansion potential [6]. Indeed, the M-MSCs used in this study could be expanded for more than 30 passages without adverse genetic or functional abnormalities and exhibited several typical MSC features, including fibroblast-like morphology and expression of surface markers characteristic to MSCs (CD73 and CD105) or pericytes (PDGFRB, CD146, and NG2), and chondrogenic, osteogenic, and adipogenic differentiation ability [9]. More importantly, M-MSCs showed superior therapeutic efficacy and long-term in vivo engraftment to adult-tissue counterparts in several animal models of interstitial cystitis/bladder pain syndrome (IC/BPS) [9, 10, 13], ketamine cystitis [11], and diabetes mellitus associated DUA, as well as asthma [33, 35]. Moreover, hESC-derived M-MSCs do not need tissue biopsy or sampling from subjects in a clinical setting, so biopsy-related adverse events can be avoided. In the present study, we demonstrate that hESC-derived M-MSCs can be also effective for treating DUA induced by chronic vascular endothelial damage.

In several preclinical and clinical trials using adult-tissue derived MSCs, poor engraftment and survival of the transplanted cells under in vivo conditions have impeded transferring MSC therapy into clinical practice. The preceding data reporting advantages of M-MSCs might be attributable to enhanced in vivo engraftment and survival [9, 10]. In the CBI-injured bladders, hB2MG^+^ engrafted cells were detected 7 days after transplantation, mainly located between the muscle and serosa of the bladder. The engrafted M-MSCs contributed little to the α-SMA^+^ myocyte. Instead, they were engrafted into pericytes or stromal cells near blood vessels and muscle fibers, suggesting that their prolonged paracrine effects repair the damaged muscle fibers. The several trophic factors secreted by MSCs are responsible for paracrine effects by mediating immune-suppressive, anti-inflammatory, and pro-angiogenic responses, which have significantly contributed to the beneficial outcomes of MSC therapy targeting several diseases. Gene expression analysis revealed that M-MSCs, compared with the differentiated fibroblast cell line, up-regulated the expression of several angiogenic genes (e.g., *VEGFA*, *PDGF-A*, and *FGF2*) and tissue regenerating WNT family genes (e.g., *WNT2B* and *WNT4*). WNT signaling plays a key role in angiogenesis and vascular remodeling or maturation in the tissue development, homeostasis and repair processes [36–39]. In particular, the secreted Frizzled-related protein-1, a modulator of the WNT pathway, stimulates the angiogenic functions of MSCs, leading to vessel maturation and functionality. Therefore, further in-depth characterization of the paracrine factors of M-MSCs, including WNT related factors, could advance our understanding of the mode of action of M-MSC therapy targeted to DUA. In addition, further study is warranted to investigate the underlying mechanisms and key player(s) that modulate the perivascular engraftment of M-MSCs in the DUA pathological condition.

To date, the pathophysiology of CBI-induced DUA has not been fully elucidated [40, 41]. In the present study, transcriptome analysis of CBI-injured bladders was characterized by the alternation of the complement system (e.g., *Cfb*, *Cfh*, and *C2*), and the inflammatory (e.g., *Csf3r*, *Cxcl-3*, and *Il-33*), ion transport (*Tf*), IL-17 (*Nfkbia*, *Cxcl2*, and *S100a9*), and HIF-1 (*Vegfa* and *Angpt-1*) signaling pathways. In response to CBI damage, VEGF and ANGPT-1 signaling can cross-talk to stimulate NF-κB activation, enhancing leukocyte-endothelial adhesion and aggravating vascular endothelial damage (Suppl. Fig. 3). Furthermore, inflammatory signals, including interleukin-1, can activate VEGF-A expression and angiogenesis in the tumor micro-environments [42–44]. Since M-MSC therapy effectively restored the dysfunction of genes related to VEGF-A and NF-κB signaling, a further study is required to determine whether the interplay between these signaling pathways is involved in CBI-induced DUA. It would also be interesting to further investigate the role of the complement system in the pathophysiology of DUA, which is relatively unknown.

The main limitation of current study is its preclinical design. The pathophysiology of DUA is multifactorial and our CBI model might not reflect the entire and complex pathogenesis. In addition, despite the promising results from this preclinical study, one major obstacle to the therapeutic application of hESC-derivatives is the safety issues, including the possible formation of teratoma or other tumors, immune rejection, and the risk that the cell will differentiate into unwanted cell types [45]. However, recent successful clinical studies for the therapy of eye disorders could alleviate this general concern of hESC-based therapeutics [46, 47]. Likewise, the aforementioned adverse outcomes were not detected in long-term monitoring up to 1 year in both acute and chronic IC/BPS animal models treated with M-MSC therapy [9, 10]. However, safety issues surrounding hESC-based therapies must still be thoroughly investigated before clinical application of these cells [45].

In conclusion, our findings provide an in vivo proof of concept for treating CBI-induced DUA with hESC-derived M-MSCs that restore bladder voiding functions, contractibility, and histological features. Further, we optimized dosage and elucidated the underlying molecular mechanisms of M-MSC therapy.

## Supplementary Information

ESM 1 (DOCX 1311 kb)

ESM 2 (XLSX 34 kb)

## Data Availability

The transcriptome data described in this study have been deposited in the Gene Expression Omnibus (GEO) of the NCBI and are accessible under GEO series accession number GSE122060 [19].
