# Supplementary material for: A Preclinical Study of Human Embryonic Stem Cell-Derived Mesenchymal Stem Cells for Treating Detrusor Underactivity by Chronic Bladder Ischemia
Source: Stem Cell Rev Rep. 2021 Jun 29;17(6):2139–52. doi: 10.1007/s12015-021-10204-z (PMC8599399; doi:10.1007/s12015-021-10204-z)
Supplement: Supplementary file 1 — (DOCX 1311 kb) [file 12015_2021_10204_MOESM1_ESM.docx]

**Supplementary Information**

**A preclinical study of human embryonic stem cell-derived mesenchymal stem cell for treating detrusor underactivity by chronic bladder ischemia**

Hwan Yeul Yu^1,2†^, Jung Hyun Shin^1†^, HongDuck Yun^2†^, Chae-Min Ryu^1,2^, Seungun Lee^2^, Jinbeom Heo^2^, Jisun Lim^2^, Juhyun Park^1^, Ki-Sung Hong^3,4^, Hyung-Min Chung^3,4^, Dong-Myung Shin^2*^, Myung-Soo Choo^1*^

*Corresponding author. d0shin03@amc.seoul.kr or mschoo@amc.seoul.kr

**Supplemental Figure Legends**

**
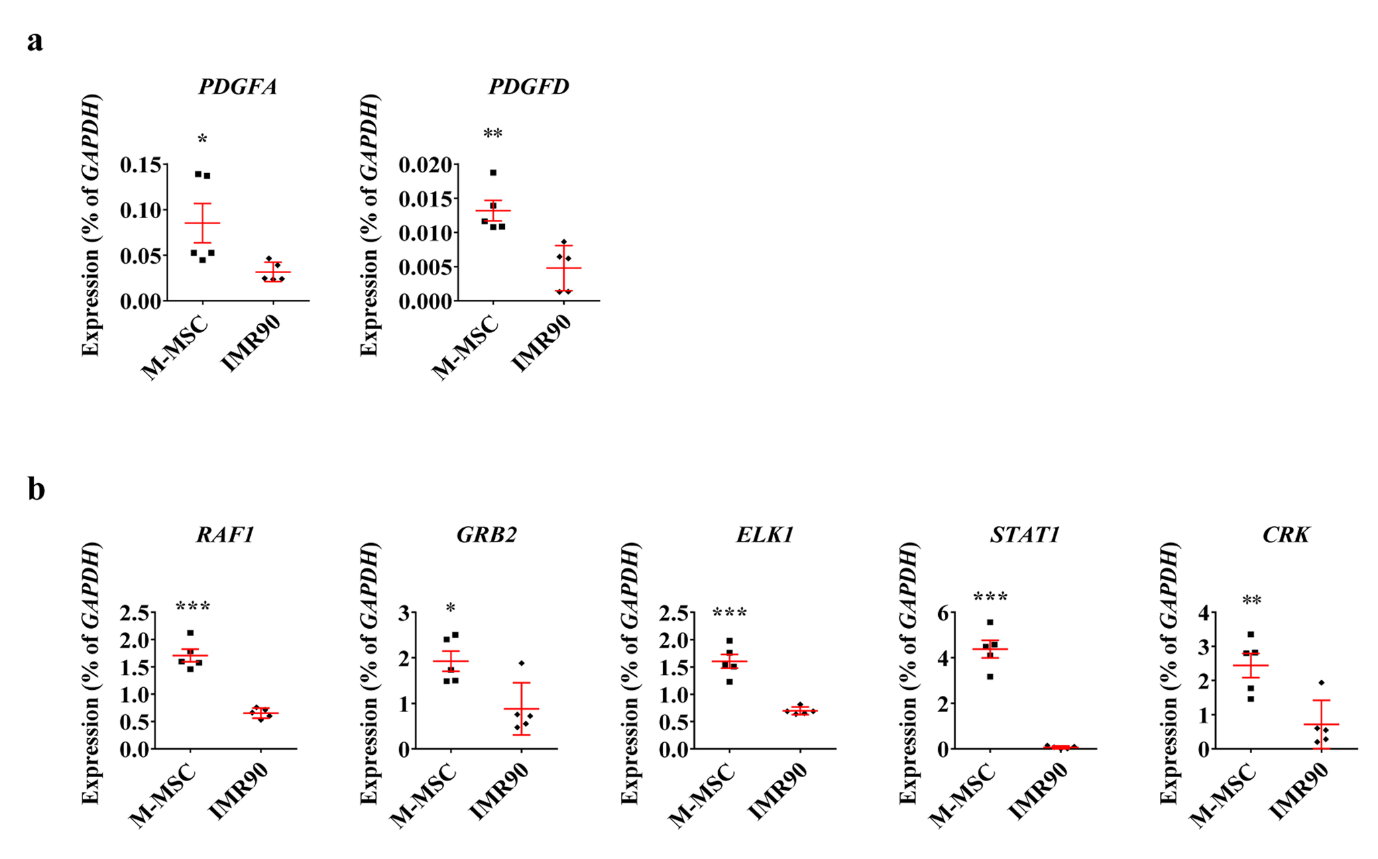
**

**Supplementary Fig. 1 Gene expression analysis of M-MSC therapy in the CBI bladders.**

RQ-PCR analysis of genes involved in the angiogenesis **(a)** and the related signaling pathways **(b)** in the M-MSCs and IMR90, a human primary lung fibroblast cell. Expression is presented as % *GAPDH* and shown as a dot plot of mean ± SEM (n=5). *p<0.05, **p<0.01, ***p<0.001 compared by a non-parametric Mann–Whitney *U* test.

**
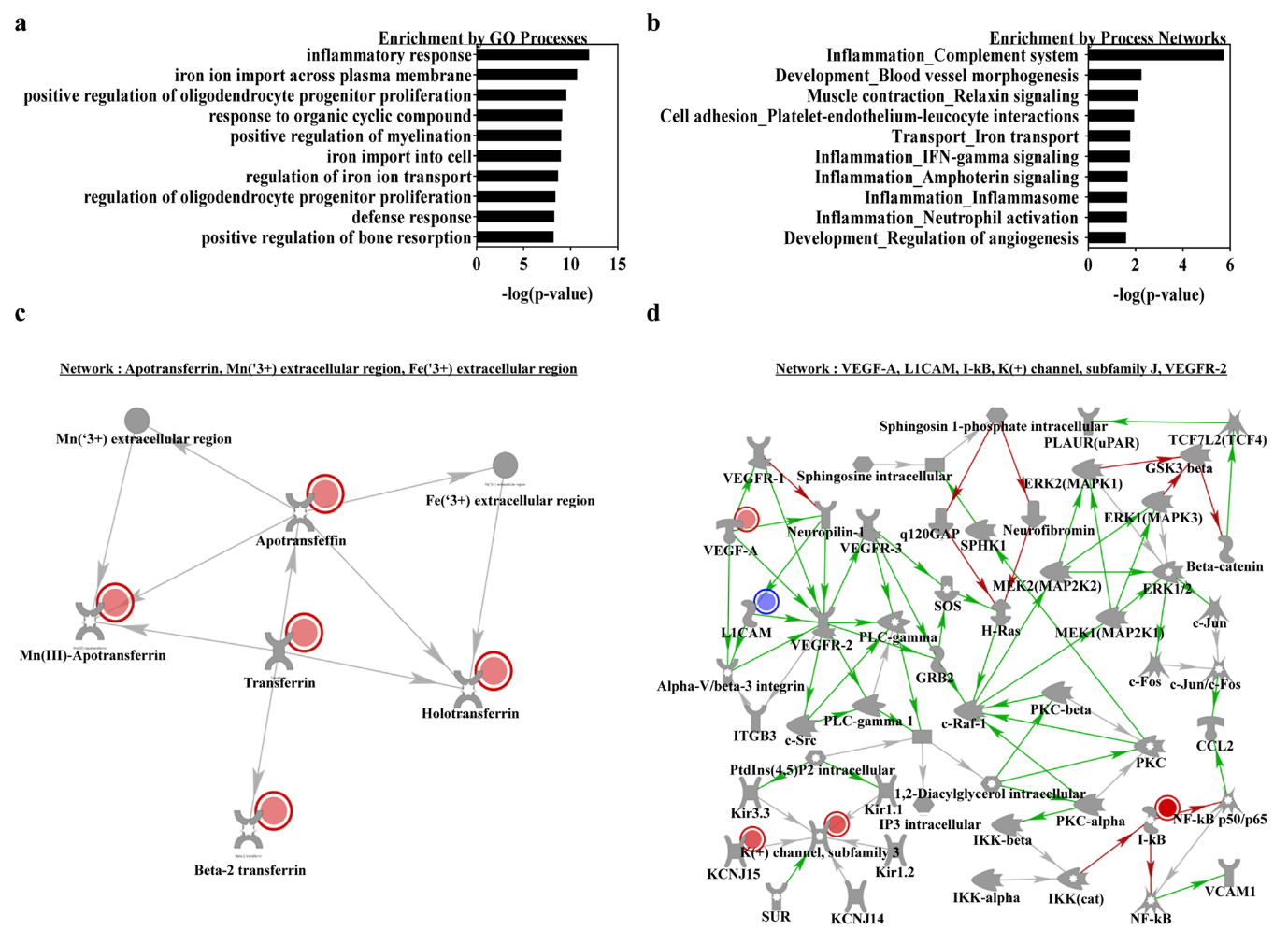
**

**Supplementary Fig. 2 Gene expression profiles of the CBI bladders.**

**(a and b)** The top Gene Ontology (GO) processes **(a)** and process networks **(b)** as revealed by MetaCore analysis comparing CBI and sham bladders. **(c and d)** Two representative gene networks related to iron ion import **(c)** or VEGF- and NFκB-mediated cellular response to growth factor stimulus **(d)**, as determined by MetaCore analysis. Gene networks are illustrated by overlaying the experimental values as fold changes in CBI versus sham samples. Up- and down-regulated genes are indicated in red and green, respectively.

**
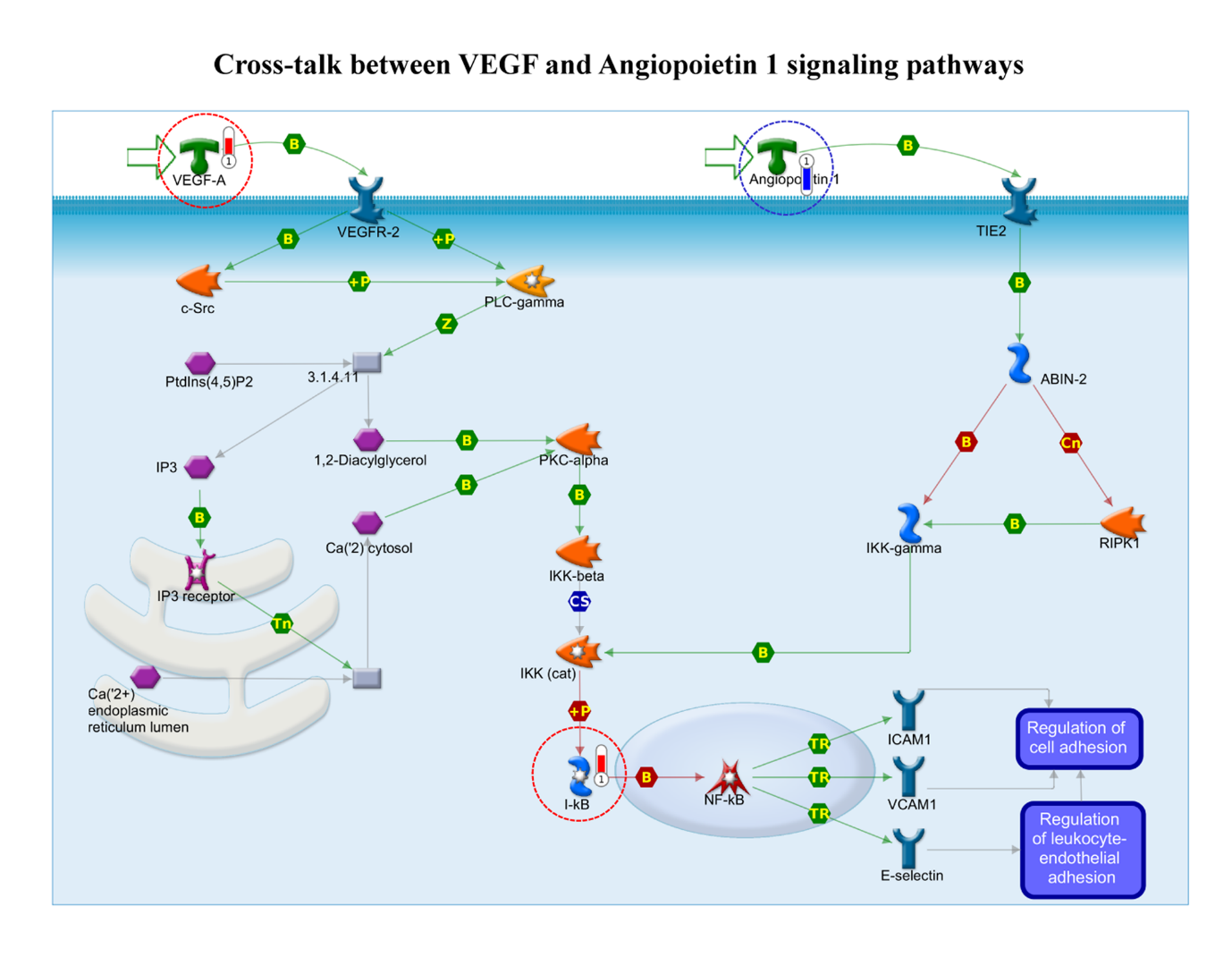
**

**Supplementary Fig. 3 Gene expression profiles of the CBI bladders.**

A schematic overview of the cross-talk between VEGF and angiopoietin 1 signaling pathways (**Fig 5b**) in MetaCore analysis of the CBI bladder transcriptome dataset. The gene expression fold changes in CBI versus sham samples are overlaid in the pathway map. Up- and down-regulated genes are indicated in red and green, respectively.
